# Dermatological Diseases Associated with Pregnancy: Pemphigoid Gestationis, Polymorphic Eruption of Pregnancy, Intrahepatic Cholestasis of Pregnancy, and Atopic Eruption of Pregnancy

**DOI:** 10.1155/2015/979635

**Published:** 2015-11-02

**Authors:** Christine Sävervall, Freja Lærke Sand, Simon Francis Thomsen

**Affiliations:** ^1^Department of Dermatology, Bispebjerg Hospital, 2400 Copenhagen NV, Denmark; ^2^Center for Medical Research Methodology, Department of Biomedical Sciences, University of Copenhagen, 2200 Copenhagen N, Denmark

## Abstract

Dermatoses unique to pregnancy are important to recognize for the clinician as they carry considerable morbidity for pregnant mothers and in some instances constitute a risk to the fetus. These diseases include pemphigoid gestationis, polymorphic eruption of pregnancy, intrahepatic cholestasis of pregnancy, and atopic eruption of pregnancy. This review discusses the pathogenesis, clinical importance, and management of the dermatoses of pregnancy.

## 1. Introduction

Three general categories of skin conditions occur during pregnancy. First, a number of benign skin conditions can occur because of normal physiological/hormonal changes during pregnancy; second, preexisting skin conditions can show signs of flare (or quiescence) during pregnancy due to immune-hormonal alterations, and third, several pregnancy-specific dermatoses can occur [1].

Pregnancy-specific dermatoses represent a group of pruritic skin diseases unique to pregnancy. Due to the not fully elucidated etiopathogenesis, the rarity of these diseases, and the clinical overlap between them, there is an ongoing discussion on how to classify the specific diseases. The latest classification is proposed by Ambrus-Rudolph [2] in 2006 and includes pemphigoid gestationis (PG), polymorphic eruption of pregnancy (PEP), intrahepatic cholestasis of pregnancy (ICP), and atopic eruption of pregnancy (AEP) (Table 1).

The purpose of this review is to elucidate these four pregnancy-specific dermatoses and their clinical importance. Some of the diseases are correlated with fetal risks, making pregnancy-specific dermatoses an important topic for the clinician. Several other dermatological diseases exist, which can be associated with pregnancy. However, these are not dealt with herein.

## 2. Pemphigoid Gestationis (PG)

PG is a rare and intensely pruritic autoimmune skin disorder that only occurs in association with pregnancy. In terms of clinical and immunologic features it is similar to the pemphigoid group of autoimmune blistering skin disorders.

PG was formerly termed herpes gestations, because of the similar morphology of the blisters. The name was changed as PG was shown not to be related to, or associated with, any prior or active herpes virus infection. The disease commonly presents in the second or third trimester of pregnancy [2, 3] but cases have also been reported in the first trimester and postpartum period [2, 4, 5]. The incidence is approximately 1 in 60.000 [6, 7] pregnancies and the disease shows a worldwide distribution. The pathogenesis is not yet fully established but an association with the haplotypes HLA-DR3 in 61–80% of patients and HLA-DR4 in 52-53% of patients has been delineated [8].

### 2.1. Pathogenesis

In PG the first immune response is located within the placenta. Circulating complement-fixing IgG antibodies develop that react with the amniotic epithelium of placental tissues and the basement membrane of the skin. The autoimmune response in the skin consists of deposition of immune complexes, complement activation, consecutive chemoattraction of eosinophils, and degranulation, resulting in tissue damage and blister formation [9]. The underlying factor that initiates the process remains unclear, but a theory proposes that an allogeneic or autoimmune response to an abnormal MHC class TI product expression in the placenta is important [10].

PG is also reported to exacerbate or flare during menstruation, or following administration of postpartum oral contraceptives. These observations suggest that changes in sex hormones might play a role in the pathogenesis of PG [6, 11, 12], although this is not congruent with some studies [13].

### 2.2. Clinical Features

**Figure 1:**
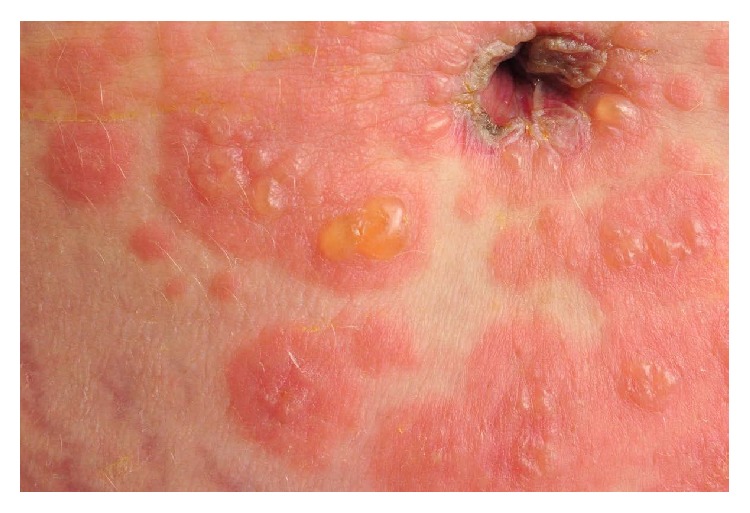
Periumbilical eruption in PG.

Initially the disease presents with pruritic urticarial papules and annular plaques, followed by vesicles and finally large tense bullae on an erythematous background. The most common eruption site is the periumbilical area (Figure 1). In 90% of the cases, it later spreads to the rest of the abdomen and, in some cases, even to thighs, palms, and soles [11]. During the last month of pregnancy the patient experiences a remission commonly followed by a flare immediately after delivery. The activity of PG decreases and often disappears during the first months after delivery but will often return in subsequent pregnancies. The disease is self-limiting and most patients exhibit a spontaneous remission in weeks to months after delivery, even without treatment.

### 2.3. Diagnosis

The diagnosis of PG relies on the clinical evaluation, histological findings, and direct immunofluorescence (DIF). The classic histologic picture shows urticarial lesions with superficial and deep perivascular lymphohistiocytic eosinophil infiltration. DIF shows a linear deposition of IgG and C3 complement at the basement membrane antigenic zone [4, 11]. C3 is reported in up to 100% of cases, while IgG is seen in 25 to 50% [11].

### 2.4. Treatment

The treatment is oral corticosteroids with a daily dose of 0.5 mg/kg, gradually tapered to a maintenance dose depending on the activity of the disease. For mild disease the use of class III or IV topical steroids can be sufficient. If topical and oral corticosteroid treatment is insufficient, systemic immunosuppressants such as cyclosporine A, dapsone, azathioprine, or methotrexate (postpartum) might be beneficial.

### 2.5. Fetal Concerns

**Figure 2:**
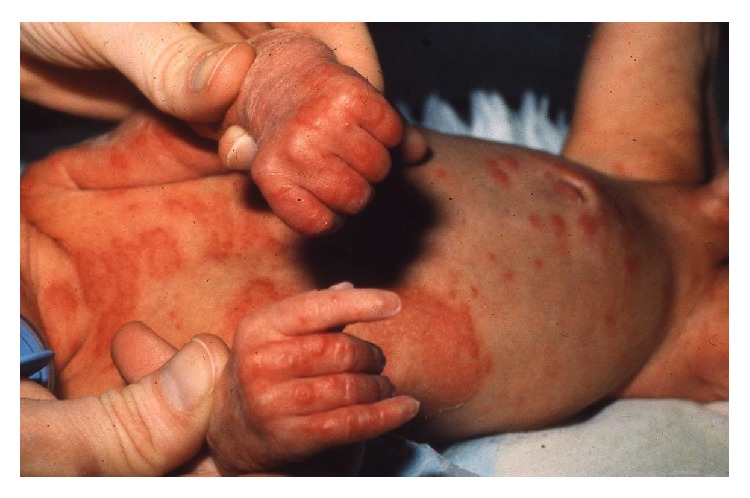
Urticaria-like and vesicular skin lesions in neonatal PG.

PG is associated with several risks for the fetus. Because of the passive transfer of IgG1 antibodies from the mother to the fetus, approximately 10% of newborns develop a mild clinical picture consisting of urticaria-like or vesicular skin lesions [9] (Figure 2). There is also a risk for premature birth and small-for-gestational-age babies. Some have theorized that systemic use of corticosteroids during pregnancy might increase the risk of developing fetal abnormities, but this is probably associated with the activity of the disease rather than the systemic use of corticosteroids. The fetal risks are especially found related to the onset of PG in the first or second trimester. The presence of blisters and/or the systemic prednisolone treatment did not appear to affect, or worsen, pregnancy outcomes in women with PG [14]. However, adverse effects of topical and systemic corticosteroids in the mother should be monitored. Also, toxicity in the mother and risk of premature birth and small-for-gestational-age babies related to cyclosporine A should be monitored closely. Azathioprine can be used during pregnancy but toxicity related to the mother should be monitored. Methotrexate is contraindicated during pregnancy.

### 2.6. Comorbidities

PG is often found in association with other autoimmune diseases such as Graves' disease, thyroiditis, and pernicious anemia [5, 11]. This can be partially explained by the presence of HLA-DR3 and DR4 [15] in both PG and these autoimmune diseases.

## 3. Polymorphic Eruption of Pregnancy (PEP)

PEP (earlier termed pruritic urticarial papules and plaques of pregnancy, PUPPP) is a benign, self-limiting inflammatory disorder that usually affects* primigravida* in the third trimester of pregnancy or immediately in the postpartum period [9, 16, 17]. It rarely recurs in subsequent pregnancies [17]. It is the most common pregnancy-specific dermatosis with an incidence of 1 in 160 pregnancies [9, 18]. Despite the relatively high frequency of PEP, little is known about its etiology. It is suggested that changes in sex hormones and immunologic responses to abdominal distension may trigger PEP, but none of these theories are substantiated [3, 9, 18].

### 3.1. Pathogenesis

The pathogenesis is still unknown and not sufficiently elucidated.* The distension theory* suggests that an overdistension of the abdominal wall causes subsequent damage to the connective tissue triggering an inflammatory response [9, 18]. A study of 200 patients found a statistically significant reduction in serum cortisol among patients with PEP [3] compared to controls, but the relevance of this is still unclear. Another theory suggests that PEP might be connected to atopy, after a study of 181 patients with PEP revealed a frequency of atopy among 55% of the included patients [18]. So far it has not been possible to find evidence of circulating immune complexes or specific HLA associations for PEP, so the pathogenic mechanisms remain unknown.

### 3.2. Clinical Features

**Figure 3:**
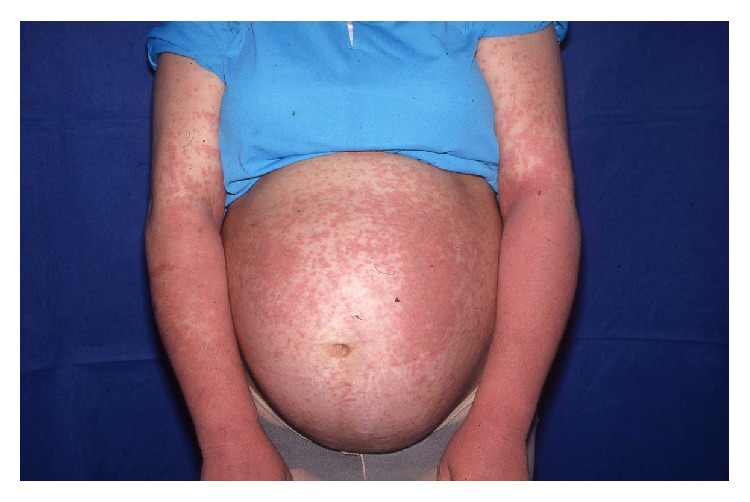
Pruritic urticarial rash in PEP.

The site of onset of symptoms is usually the abdomen, often within* striae distensae* and with an intensely pruritic urticarial rash with erythematous, edematous papules, and plaques (Figure 3). Sparing of the umbilical region is a characteristic finding. The disease spreads to other body sites such as proximal thighs, buttocks, and the back. Rarely the eruption spreads ideally involving arms and legs [9]. The morphology changes while the disease advances, developing polymorphic features such as papulovesicles, erythema, and annular wheals.

### 3.3. Diagnosis

There are no tests that definitely can decide whether a woman has PEP. DIF and indirect immunofluorescence are negative in PEP. The histopathology varies with the stage of the disease. The diagnosis is based on the clinical picture, with the abovementioned characteristics, and a biopsy showing dermal edema, a superficial to mid-dermal perivascular lymphohistiocytic infiltrate composed of eosinophils, T-helper cells, and macrophages. At a later stage a biopsy reveals epidermal changes including hyper- and parakeratosis [9, 16].

### 3.4. Treatment

Treatment is symptomatic. In order to control pruritus and advancement of the skin rash it is usually sufficient to use topical corticosteroids with or without oral antihistamines, but in severe cases a short treatment burst with systemic corticosteroids may be necessary [9, 16]. The disease is self-limiting and the lesions usually resolve within weeks after birth, with no postinflammatory pigmentary change or scarring.

### 3.5. Fetal Concerns

PEP is not associated with cutaneous manifestations or risk to the newborn or fetus. The maternal prognosis is excellent in most cases [18]. Maternal adverse effects of systemic and topical corticosteroids should be monitored. Not all antihistamines are approved during pregnancy; cetirizine, loratadine, and fexofenadine should be preferred.

## 4. Intrahepatic Cholestasis of Pregnancy (ICP)

ICP, also known as pruritus gravidarum, is a liver disorder characterized by severe pruritus and secondary skin lesions in the third trimester of pregnancy. The symptoms develop from a reversible form of hormonally triggered cholestasis that typically develops in genetically predisposed individuals. ICP is not a primary dermatosis, but due to its correlation with fetal risks and skin symptoms, it is regarded as a pregnancy-specific dermatosis. The prevalence of ICP is around 1% [9, 16, 19, 20] but it shows a striking geographical pattern with a higher prevalence in Scandinavia and South Africa.

### 4.1. Pathogenesis

The pathogenesis is multifactorial involving an interaction between hormonal changes (being the main factor), genetic predisposition, and exogenous factors [9]. Exogenous factors include environmental factors such as seasonal variability [21] and dietary factors such as decreased selenium levels [22]. The role of the exogenous factors is still debated.

### 4.2. Clinical Features

**Figure 4:**
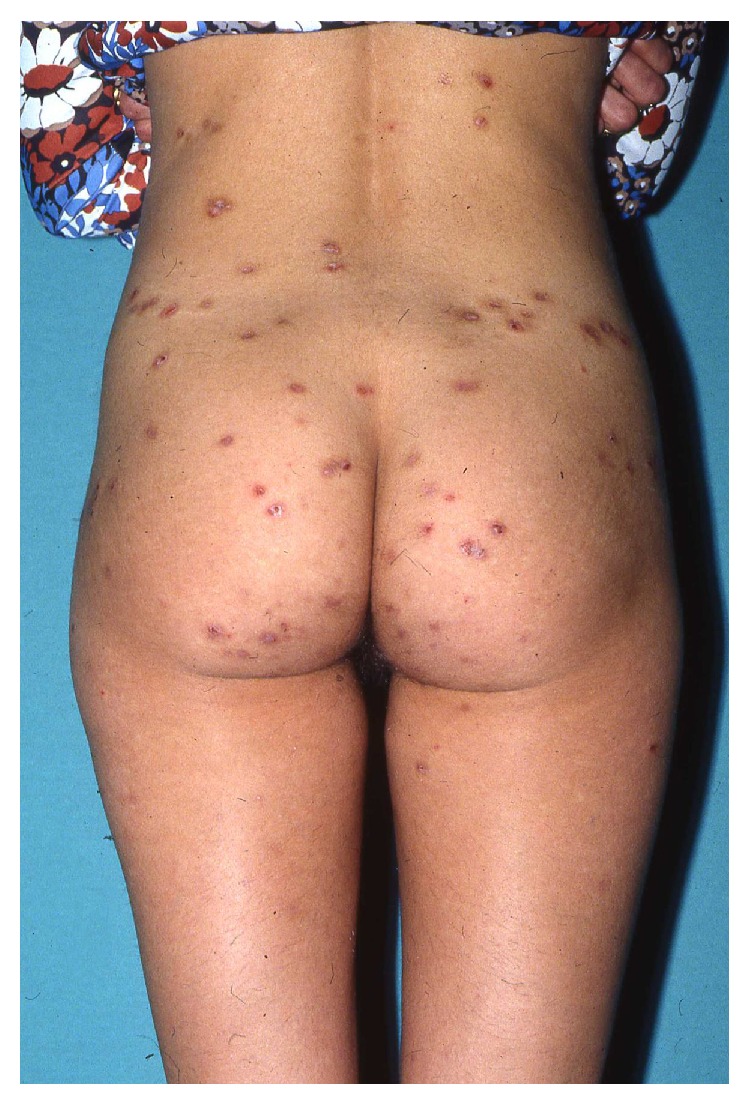
Excoriations, scratch marks, and prurigo nodules in ICP.

ICP is characterized by severe pruritus with no primary skin lesions with or without jaundice, which is seen in 0.02–2.4% [23]. Pruritus typically starts on the palms and soles and later becomes generalized. Later secondary lesions such as excoriations, scratch marks, and prurigo nodules might develop as a result of scratching (Figure 4). This commonly involves the shins and lower arms. The symptoms usually disappear 1-2 days after delivery, but in some cases they can persist for 1-2 weeks [9]. There is a high risk of recurrence of ICP in subsequent pregnancies (50–70%) and with the use of oral contraceptives.

### 4.3. Diagnosis

The hallmarks for diagnosing ICP are the generalized pruritus and the elevated serum bile acid levels and aminotransferases.

### 4.4. Treatment

Treatment aims to lower the level of serum bile acid and to alleviate pruritus. Ursodeoxycholic acid can be used to alleviate the severity of pruritus and has shown to give a more favorable outcome of pregnancy and the absence of adverse events [24]. Treatments with cholestyramine, antihistamines, and oral corticosteroids have been tried, but none are supported by current evidence or may have adverse effects [1, 24]. Phototherapy with UVB can be used in refractory cases.

### 4.5. Fetal Concerns

ICP is correlated with fetal risks, the most common being premature birth (20–60%) followed by intrapartal fetal distress (20–30%) and stillbirth (1-2%) [9]. In severe or prolonged ICP, cholestasis might cause vitamin K deficiency and coagulopathy in patients and their children [1]. These risks make it necessary to closely follow these during and after pregnancy.

## 5. Atopic Eruption of Pregnancy (AEP)

AEP is a benign pruritic condition that is characterized by eczematous or papular lesions in patients with a history of, or predisposition to, atopic dermatitis or with new onset of atopic dermatitis during pregnancy. The term AEP covers a heterogeneous group of pruritic conditions during pregnancy also known as prurigo of pregnancy, pruritic folliculitis of pregnancy, and eczema in pregnancy. AEP is the most common cause of pruritus during pregnancy [2, 16] with a prevalence of 5–20%. AEP includes two groups of patients, one who during pregnancy either experience atopic skin changes for the first time or after a long remission and, second, patients who suffer from an exacerbation of preexisting atopic dermatitis. A study of 505 women showed that 80% experienced skin changes for the first time [2]. The symptoms usually start early in the first or second trimester and typically reoccur in subsequent pregnancies due to the atopic background. Many women with AEP have elevated serum IgE, a positive allergy test for airborne allergens, and a family history of atopic diseases.

### 5.1. Pathogenesis

The pathogenesis of AEP and the late onset of symptoms are thought to be triggered by pregnancy-specific immunological changes. During pregnancy, women have an altered pattern of T-helper (Th) cells with a reduced production of Th1 cytokines (IL-2, interferon gamma, and IL-12) and an increased Th2 cytokine (IL-4 and IL-10) production [25]. The Th2 response is thought to be responsible for the skin changes seen in pregnant women.

### 5.2. Clinical Features

**Figure 5:**
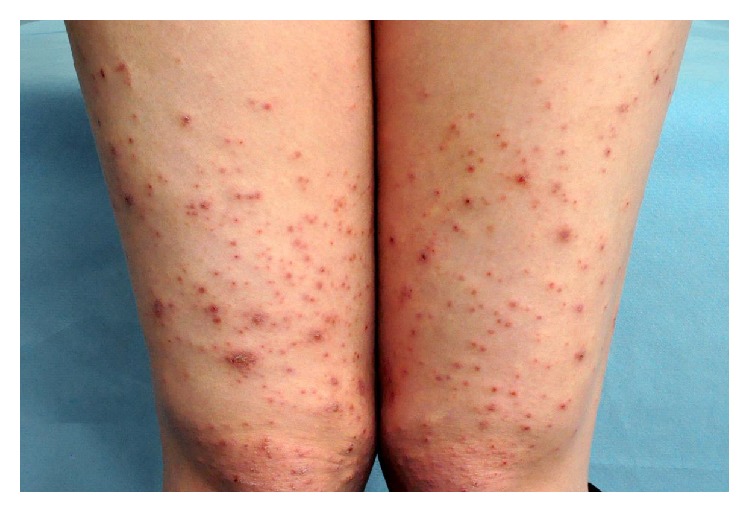
Prurigo lesions/excoriations and eczematous-like skin lesions in AEP.

The main clinical features are pruritus, prurigo lesions/excoriations, and eczematous-like skin lesions (Figure 5). Two-thirds present with widespread eczematous changes affecting typical atopic sites such as the face, neck, and the flexor surfaces of the extremities, while one-third have small pruritic, erythematous papules on the trunk and limbs. Scratching causes excoriations and might result in secondary skin infections. The eczema usually disappears after pregnancy.

### 5.3. Diagnosis

The diagnosis is mostly based on the clinical characteristics. There are no pathognomonic findings specific to AEP, but laboratory tests might reveal elevated serum IgE levels in 20–70% [2].

### 5.4. Treatment

Treatment strategy depends on the severity of the condition. The treatment is topical corticosteroids class III or IV. This is usually sufficient, but in severe cases systemic corticosteroids or antihistamines may be required. UVB phototherapy is used for recalcitrant cases. In case of secondary bacterial infection with hemolytic streptococci or staphylococci, treatment with antibiotics is necessary.

### 5.5. Fetal Concerns

AEP is not associated with fetal risks, except the uncertain risk for the child to develop atopic dermatitis.

## 6. Conclusion

Pruritus and skin changes are common during pregnancy and are usually benign and self-limiting. In some cases, however, they are symptoms of pregnancy-specific dermatoses. These constitute a rare group of inflammatory dermatoses specifically related to pregnancy and/or the immediate postpartum period, which can be associated with severe fetal outcomes such as fetal distress, stillbirth, and premature birth [23].

Pruritus represents the leading symptom in this group of diseases. Skin changes vary in morphology, location, and time of onset, but still there are many similarities. For the untrained eye it might be difficult to separate the different diagnoses by only using clinical characteristics. Direct immunofluorescence, histopathology, and blood analyses are used as complementary diagnostic tools for a more correct diagnosis. Only for PG and ICP laboratory tests can substantiate the clinical diagnosis. Therefore, it is important for the clinician to combine the medical history, the morphologic criteria, and the histopathology of the lesions to establish the correct diagnosis.

Fetal risks have only been associated with PG and ICP, but with the overlapping symptoms between the diseases pruritus in pregnancy should never be neglected. Interdisciplinary management involving dermatologists, pediatricians, obstetricians, and gastroenterologists is mandatory to acquire a better outcome for the mother and the fetus.

## Figures and Tables

**Table 1:** 

Pregnancy dermatosis	Suggested pathogenesis	Clinical features	Localisation	Paraclinical diagnosis	Treatment	Fetal concerns
Pemphigoid gestationis (PG)	Complement-fixing IgG antibodies and complement C3 react with the amniotic epithelium of placental tissues and the basement membrane of the skin causing an autoimmune response resulting in tissue damage and blister formation	Pruritic urticarial papules and annular plaques followed by vesicles and finally large tense bullae on an erythematous background	Eruption site is the periumbilical area (most common), rest of the abdomen, thighs, palms, and soles	Histology: urticarial lesions with superficial and deep perivascular lymphohistiocytic eosinophil infiltration DIF: linear deposition of IgG and C3 complement at the BMZ	Oral corticosteroids at a daily dose of 0.5 mg/kg gradually tapered to a maintenance dose depending on the activity of the disease Classes III-IV topical steroids. Cyclosporine A, dapsone, azathioprine, or methotrexate (postpartum)	Passive transfer of IgG1 antibodies can cause mild urticaria-like or vesicular skin lesions in newborns Risk for premature birth and small-for-gestational-age babies Risk of small-for-gestational-age and preterm birth with cyclosporine A Drug toxicity should also be monitored in the mother

Polymorphic eruption of pregnancy (PEP)	Abdominal distension causing subsequent damage to the connective tissue triggering an inflammatory response Differences in cortisol level in patients with PEP Connection to atopy	Intensely pruritic urticarial rash with erythematous, edematous papules, and plaques, developing into polymorphic features such as papulovesicles, erythema, and annular wheals	Onset on the abdomen with sparing of the umbilical region as a characteristic finding, which later spreads to thighs, buttocks, and back	Histology: dermal edema with a superficial to mid-dermal perivascular lymphohistiocytic infiltrate composed of eosinophils, Th cells, and macrophages	Topical corticosteroids and oral antihistamines Oral corticosteroids	No adverse effects related to PEP Prednisone and prednisolone do not readily cross the placenta and can be safely used during pregnancy Drug toxicity should be monitored in the mother Only certain antihistamines approved during pregnancy

Intrahepatic cholestasis of pregnancy (ICP)	Hormonal changes Genetic predisposition Exogenous factors (seasonal variability and dietary factors)	Severe pruritus with no primary skin lesions occurring with or without jaundice	Onset on palms and soles to later become generalized Secondary lesions such as excoriations, scratch marks, and prurigo nodules might develop	Elevated serum bile acid levels (and aminotransferases)	Ursodeoxycholic acid to alleviate the severity of pruritus and to give a more favorable outcome of pregnancy and the absence of adverse events UVB Phototherapy	Premature birth Intrapartal fetal distress Stillbirth Vitamin K deficiency and coagulopathy in the mother and newborn

Atopic eruption of pregnancy (AEP)	Altered pattern of Th cells with a reduced production of Th1 cytokines (IL-2, interferon gamma, and IL-12) and an increased Th2 cytokine (IL-4 and IL-10) production	Pruritus, prurigo lesions/excoriations, and eczematous-like skin lesions Secondary infection due to excoriations	66% present with widespread eczematous changes affecting typical atopic sites 33% have small pruritic, erythematous papules on the trunk and limbs	No pathognomonic findings specific to AEP Elevated serum IgE levels in 20–70%	Topical corticosteroids classes II–IV Oral corticosteroids and antihistamines UVB phototherapy Antibiotics in cases of secondary infection	No adverse effects except the uncertain risk for the child to develop atopic dermatitis

DIF: direct immunofluorescence; BMZ: basement membrane zone; Th: T-helper.
